# The effect of implicit racial bias on recognition of other-race faces

**DOI:** 10.1186/s41235-021-00337-7

**Published:** 2021-10-30

**Authors:** Tobiasz Trawiński, Araz Aslanian, Olivia S. Cheung

**Affiliations:** Division of Science, Department of Psychology, New York University, Abu Dhabi, UAE

**Keywords:** Implicit association test, Eye movements, Face recognition, Other-race effect

## Abstract

Previous research has established a possible link between recognition performance, individuation experience, and implicit racial bias of other-race faces. However, it remains unclear how implicit racial bias might influence other-race face processing in observers with relatively extensive experience with the other race. Here we examined how recognition of other-race faces might be modulated by observers’ implicit racial bias, in addition to the effects of experience and face recognition ability. Caucasian participants in a culturally diverse city completed a memory task for Asian and Caucasian faces, an implicit association test, a questionnaire assessing experience with Asians and Caucasians, and a face recognition ability test. As expected, recognition performance for Asian faces was positively predicted by increased face recognition ability, and experience with Asians. More importantly, it was also negatively predicted by increased positive bias towards Asians, which was modulated by an interaction between face recognition ability and implicit bias, with the effect of implicit bias observed predominantly in observers with high face recognition ability. Moreover, the positions of the first two fixations when participants learned the other-race faces were affected by different factors, with the first fixation modulated by the effect of experience and the second fixation modulated by the interaction between implicit bias and face recognition ability. Taken together, these findings suggest the complexity in understanding the perceptual and socio-cognitive influences on the other-race effect, and that observers with high face recognition ability may more likely evaluate racial features involuntarily when recognizing other-race faces.

## Introduction

The ideal socio-cultural norms promote anti-discrimination behavior. However, although there is an increasing amount of anti-discrimination policies implemented at various levels of the society, the biases against individuals of other races have not been decreased in recent years (Wetts & Willer, 2018). There is a large body of evidence that individuals in minority racial groups are discriminated against during hiring processes (Zschirnt & Ruedin, 2016) or medical treatments (Goyal et al., 2015), and they also often receive suboptimal housing or credits conditions (Pager & Shepherd, 2008). During various everyday activities, face misidentification may have serious consequences for eyewitness testimony (Sporer, 2001), border control (Bobak et al., 2016), or identification of the perpetrator of the crime (Anwar et al., 2012), especially for individuals of other races. Understanding the factors that contribute to the successful recognition of other-race faces may help to produce solutions to alleviate some adverse effects of racial issues in social behavior.

The other-race effect (ORE), which is revealed by poorer recognition of other-race faces compared with own-race faces, has been consistently observed (for a review, see Meissner & Brigham, 2001). The poor recognition of other-race faces is thought to be associated with differential processing strategies for own- vs. other-race faces, particularly impaired holistic processing for other- than own-race faces (Hayward et al., 2013; Michel et al., 2006; Mondloch et al., 2010; Tanaka et al., 2004), or speeded categorization of the other-race faces by race instead of recognizing them as individuals (Levin, 1996, 2000). The main contributors to the ORE include perceptual and socio-cognitive factors (Hugenberg et al., 2010; Meissner & Brigham, 2001; Sporer, 2001). Specifically, the lack of contact experience with individuals of the other race (Allport, 1954; Chiroro & Valentine, 1995; Valentine et al., 2016; Williams, 1947), or a negative implicit bias about the other race (Walker & Hewstone, 2006, 2008), likely contribute to the ORE. Notably, these factors may not be mutually exclusive, and the relationship between perceptual and socio-cognitive factors on other-race face processing is yet to be fully understood.

The ORE appears to be particularly robust in individuals who are part of the majority population in the community (e.g., Caucasian participants in a predominantly Caucasian community) and have relatively little contact experience with individuals from another race (Chiroro & Valentine, 1995; Hugenberg et al., 2010; Sporer, 2001; Valentine et al., 2016). Although the ORE has been frequently reported, the magnitude of the ORE has been consistently reduced in the past decades, potentially due to the increase in interracial contact (Meissner & Brigham, 2001). There has been increasing evidence of a lack of the ORE in individuals who have relatively extensive experience with individuals of another race, such as Asian participants in Europe viewing Caucasian faces, or Malaysian Indians or Malays viewing Chinese faces (de Heering et al., 2010; Estudillo et al., 2019; Fioravanti-Bastos et al., 2014; Walker & Hewstone, 2006; Wright et al., 2003). Moreover, a reverse ORE has also been reported for Caucasian vs. Asian faces in Western-raised Asian observers (Sangrigoli et al., 2005). Nonetheless, the quality of the experience with another race appears to have a substantial impact on various aspects of other-race face processing. While general social contact experience is found to be positively related to memory performance of other-race faces, such experience appears to have little effect on holistic processing of other-race faces (Zhao et al., 2014). On the other hand, individuating experience appears critical to improve perceptual discrimination or enhance holistic processing of other-race faces (Bukach et al., 2012; McGugin et al., 2011; Walker & Hewstone, 2006, 2008).

Importantly, previous studies have also suggested a positive link between individuating experience and implicit racial bias, and have explored the influence of implicit racial bias on the processing of other-race faces. Implicit racial bias is defined as the unconscious attitudes towards members of another race group that cannot be directly inferred through introspective awareness (Devine, 1989; Gaertner & Mclaughlin, 1983; Greenwald & Banaji, 1995). Implicit racial biases towards members of other races can influence various judgments of other-race faces, such as categorization (Elliott et al., 2017; Hugenberg & Bodenhausen, 2004; Hutchings & Haddock, 2008), identification (Chiao et al., 2006), and attractiveness or trustworthiness judgments (Burke et al., 2013; Rhodes et al., 2005; Stanley et al., 2011). A positive implicit bias towards another race is found to be associated with enhanced perceptual discrimination performance of faces of that race (Walker & Hewstone, 2008). Moreover, training participants to individuate faces from another race, instead of categorizing them by the race, reduces negative implicit racial biases while improving memory performance of individual faces of that race (Lebrecht et al., 2009).

Today, in the age of globalization, cross-cultural and cross-racial interactions have further increased. Although increased social contact experience may lead to positive implicit racial biases towards other races, it also appears that the increased interactions could bring conflicts among individuals of different races, in terms of domestic or international issues such as immigration. Furthermore, racial issues are often divisive topics throughout different spectra of the societies, with massive influences via traditional or social media. Therefore, it is possible that the increase in individuating or social contact experience of another race might not necessarily lead to positive implicit racial bias towards that race. Instead, individuals with comparable amounts of experience with another race may show a range of implicit attitudes towards that race.

In this study, we examined the effects of implicit racial bias on recognition performance of other-race faces, while taking into account the individuating and social contact experience with the other race, and face recognition ability of the participants. While most people are generally good at recognizing faces, there is a wide range of individual differences in the ability to recognize them (DeGutis et al., 2013; Duchaine & Nakayama, 2006; Richler et al., 2011; Wang et al., 2012; Wilmer et al., 2010). It is important to note that while low recognition ability for own-race faces is a risk factor for poor recognition performance for other-race faces, the recognition ability for own-race faces does not necessarily predict the magnitude of the ORE (Wan et al., 2017). Here we examined whether interracial experience, implicit racial bias, and recognition ability for own-race faces, may have independent or interactive influences on the ORE and on recognition of other-race faces.

## Eye movements during learning of other- and own-race faces

For face recognition, eye movements are often made to specific features or regions of a face, particularly the eyes, nose, or mouth areas, or featureless regions in between the eyes and nose (Fu et al., 2012; Hills & Pake, 2013; Miellet et al., 2013; Rodger et al., 2010). Although several studies suggested that observers from different cultures, such as Caucasians and Asians, showed different fixation patterns on faces (Blais et al., 2008; Kelly et al., 2011; Liu et al., 2011), other studies did not find such differences (e.g., Caldara et al., 2010; Chuk et al., 2017; Or et al., 2015). Additionally, there have also been mixed findings on whether different fixation patterns are utilized when processing other- and own-race faces. Some studies suggested that own-race faces receive a greater number but shorter fixations than other-race faces (e.g., Goldinger et al., 2009; Wu et al., 2012), other studies showed instead that the fixation patterns were comparable for other- and own-race faces (Blais et al., 2008; Burgund, 2021; Chuk et al., 2017). Indeed, due to individual differences, observers might adopt similar or different strategies when viewing other- and own-race faces, although many observers (75%) appear to show similar overall fixation patterns when viewing other- and own-race faces (Chuk et al., 2017).

Instead of taking into account all fixations, it appears that the first two fixations on a face are highly important to achieve optimal recognition performance, as additional fixations do not significantly improve performance (Hsiao & Cottrell, 2008). The slight variations in the landing positions of the first and second fixations allow for sufficient information to be sampled for recognition, and the landing positions of the first two fixations are often at the center or towards the left side of the face (Hsiao & Cottrell, 2008; Schwedes & Wentura, 2019). The left-side bias, a tendency of fixating towards the left side of a face, appears to be related to better recognition performance (Chuk et al., 2017; Hsiao & Cottrell, 2009; van Belle et al., 2010). However, there do not appear to be significant differences, in terms of the positions of the first and subsequent fixations, between viewing own- or other-race faces, presumably because of the large variability within participants of the same race as well as across races (Or et al., 2015; Peterson & Eckstein, 2012; see also Chuk et al., 2017). Notably, it appears that the distributions of the second fixations were particularly variable among observers, compared with those of the first fixations for both other- and own-race faces (Or et al., 2015; see also Hsiao & Cottrell, 2008). Here we focused on the landing positions of the first and second fixations to other-race faces during the learning session, to examine whether individual differences in the positions of these initial fixations for other-race faces may be influenced by experience with the other race, implicit racial bias, and face recognition ability.

## The present study

To examine the effect of implicit racial bias, we tested a sample of Caucasian (White) participants in a highly multicultural community (in Abu Dhabi, UAE). In this study, the Caucasian participants had relatively extensive experience with Asian individuals, and potentially had a range of implicit attitudes towards Asian people. Using a sample with relatively extensive experience with the other race would allow us to better distinguish between the effects of experience and implicit racial bias. The study included a total of four tasks: a memory task with Asian and Caucasian faces that assessed recognition performance of other- and own-race faces, the Implicit Association Test (IAT) with Asian or Caucasian faces paired with positive or negative words that assessed implicit racial biases (Greenwald et al., 1998, 2003), the Cambridge Face Memory Test with Caucasian faces that assessed face recognition ability (CFMT, Duchaine & Nakayama, 2006), and both the social contact and individuating experience scales with the other- and own-races from Walker and Hewstone (2006). During the face memory task, we also recorded eye movements during the learning and testing sessions.

In this study, we expected to observe a range of face recognition ability and implicit racial bias scores, and relatively high experience scores with own- and other-race individuals. Importantly, we expected that different factors would predict recognition performance for own- and other-race faces. For own-race faces, recognition performance may be predicted primarily by face recognition ability of the participants only, and not by implicit biases towards another race or experience with own-race individuals, particularly because the experience scores with own-race individuals could be very high. For other-race faces, we investigated the extent that recognition performance might be explained by implicit racial bias, social contact or individuating experience with the other race, and face recognition ability of the participants. With regard to eye movements, we expected that the factors that influenced recognition performance in the memory task for other-race faces would also contribute to the variability in landing positions of the first two fixations during learning of the other-race faces.

## Method

### Participants

Fifty-three Caucasian undergraduate students (26 males and 27 females, *M* = 20.6, SD = 1.74) from the New York University Abu Dhabi (NYUAD) participated for course credits or subsistence allowance. The student population at NYUAD consists of over 1300 students from more than 120 countries in the world, and students have frequent interactions with individuals from different racial and cultural backgrounds. The participants in this study were from over 20 countries. Most of them (> 85%) were from Europe (including the UK, Macedonia, Croatia, Russia, Poland, Hungary, Germany, etc.), < 10% were from the USA, and < 5% were from Australia. All participants reported normal or corrected-to-normal vision. The final sample size was determined based on previous studies that examined individual differences between implicit racial bias and experience with other races, particularly Walker and Hewstone (2006, 2008).

### Apparatus

The stimuli were presented on a BenQ XL2411Z monitor and participants responded by pressing one of the dedicated keys on the standard keyboard. Eye movements were recorded from the left eye only, using the EyeLink 1000 Plus eye tracker from SR Research. The eye tracker was operated at 1000 Hz. Head movement was stabilized using a chin and headrest through the memory task.

### Stimuli and procedure

The experiment included a total of 4 tasks: the main memory task, Implicit Association Test (IAT), Cambridge Face Memory task (CFMT), and an experience questionnaire.

#### Memory task

In the main study, 28 Caucasian and 28 Asian faces from the Chicago Face Database (Ma & Wittenbrink, 2015) were used. The faces were selected based on race and gender. All faces were shown in frontal view with a neutral expression. For each race, the ratio of female and male faces was 50/50. An oval frame was placed around each face to cover any hair and areas beyond the chin and ears. All images were saved in grey scale. Each image was 522 pixels in height and 366 pixels in width, subtending a visual angle of 11.11° in height and 7.84° in width. According to the norming ratings from the Chicago Face Database with 7-point scales, the selected Caucasian and Asian faces were comparable in terms of unusualness (Asian: *M* = 2.02*, *SD = 0.28; Caucasian: *M* = 2.16*, *SD = 0.42; *t*_54_ = − 1.46, *p* = 0.15), attractiveness (Asian: *M* = 3.33*, *SD = 0.75; Caucasian: *M* = 3.57*, *SD = 0.75; *t*_54_ = − 1.19, *p* = 0.24), or age (Asian: *M* = 27.52*, *SD = 3.70; Caucasian: *M* = 27.75*, *SD = 4.93; *t*_54_ = − 0.20, *p* = 0.85).

The memory task was divided into two blocks, with one block of Asian faces and another block of Caucasian faces. The presentation order of the two face races was counterbalanced across participants. Within each block, there was a learning and a testing session. During the learning session, participants were asked to remember 14 faces of each race, randomly selected from each face set. Each face was shown one at a time for 5 s. Immediately after the learning session, the testing session began. During the testing session, participants were randomly shown the 14 old faces and 14 new faces, one at a time for up to 10 s each. Participants were asked to perform an “old/new” judgment task using two keys on a keyboard. All trials during learning and testing began with a fixation at the center of the screen for 500 ms, followed by a face presented in one of the four quadrants of the screen.

Eye movements were recorded during both learning and testing sessions, although the analysis focused on the learning session. Before each session, a nine-point calibration procedure was completed with the calibration of less than 0.5° error.

#### Implicit association test (IAT)

The IAT with Asian and Caucasian faces, and positive and negative words were used to measure implicit racial biases towards Asians. An additional set of 6 Asian faces and 6 Caucasian faces (3 males and 3 females for each race) from the Chicago Face Database (Ma & Wittenbrink, 2015) was used. These faces were also shown in frontal view with a neutral expression, and no oval frame was added on them. The 6 positive words were loyal, kindness, happy, trust, friend, and pleasure, and the 6 negative words were terrible, toxic, hatred, useless, brutal, and traitor.

The IAT included a total of five blocks of trials (Greenwald et al., 1998). In Block 1, either an Asian or Caucasian face was randomly presented on each trial. Participants were asked to categorize each face by race and respond on the keyboard by pressing either a key for Caucasian faces or another key for Asian faces (the key mapping was counterbalanced across participants). In Block 2, a word of either positive or negative meaning was randomly shown on each trial. Participants were asked to categorize each word by pressing one of the two keys for positive words and the other key for negative words. In Block 3, either a face (Asian or Caucasian) or a word (positive or negative) was randomly presented on each trial. Participants categorized each face or word using the assigned response keys from Blocks 1 and 2. For half of the participants, one response key was assigned for positive words and Asian faces, and another response key for negative words and Caucasian faces in Block 3. For the other half of the participants, the response key mapping in Block 3 was with a response key assigned for positive words and Caucasian faces, and another key for negative words and Asian faces. In Block 4, the procedure was identical to Block 1, except that the response keys for the different face races were switched. In Block 5, the procedure was the same as in Block 3, except that the response keys for the faces were identical to the assigned keys in Block 4. Blocks 1, 2, and 4 each had a total of 40 trials. Blocks 3 and 5 had a total of 120 trials.

The main analysis of implicit biases focused on Blocks 3 and 5. Following the improved algorithm from Greenwald et al., 2003, response times for the correct trials were calculated, with any outliers removed (< 250 ms or > 10 s) to produce a *D* score for each participant. Moreover, the first 40 trials in each of the two blocks were considered ‘practice’ trials and the remaining 80 trials were considered ‘test’ trials. The reliability of the IAT was high in the present study: Cronbach’s $\alpha$ was 0.902, and the correlations between the practice versus test trials were *r*_51_ = 0.835, *p* < 0.001 in the Asian-positive/Caucasian-negative block, and *r*_51_ = 0.827, *p* < 0.001 in the Asian-negative/Caucasian-positive block. As suggested by Greenwald et al. (2003), the practice and test trials were analyzed separately, and the scores were then averaged to form the final scores. The *D* score, which indicates the differential performance between the response key mappings between Asian-positive/Caucasian-negative and Asian-negative/Caucasian-positive, was used to reveal implicit biases. A positive *D* score indicated positive implicit bias towards Asians, whereas a negative *D* score indicated negative implicit bias towards Asians. Table 1 shows the descriptive statistics of the *D* score. While there was a range of positive and negative implicit bias scores, a two-tailed one-sample *t*-test against 0 (no bias) revealed a significant negative bias towards Asians in the sample (*t*_52_ = − 3.33, *p* < 0.001, *d* = − 0.46).

**Table 1:** The descriptive statistics for the measures of implicit racial bias (IAT), face recognition ability (CFMT), and experience with Asian and Caucasian people (N = 53)

	Mean	Median	SD	Min	Max
IAT	− 0.15	− 0.16	0.33	− 0.94	0.70
CFMT	77.65	80.56	12.50	40.28	97.22
Experience: Asian	3.15	3.10	0.77	1.50	4.70
Experience: Caucasian	4.14	4.20	0.63	2.50	5.00

#### Cambridge face memory test (CFMT)

The well-established CFMT (Duchaine & Nakayama, 2006) was used to measure face recognition ability. Across the three blocks of trials in the CFMT, participants were tested on their ability to recognize 6 unfamiliar individuals. During the initial study phase, 6 target faces were presented. During the test phase, one of the target faces appeared with 2 distractor faces on each trial. In Block 1, the test images involved identical images from the study phase. The test images involved novel images of the individuals (e.g., shown in different orientations) in Block 2, and involved novel images of the individuals with added visual noise in Block 3. There were 18, 30, and 24 trials in Blocks 1–3. The accuracy scores across the three blocks of trials were used to reveal face recognition ability. Table 1 shows the descriptive statistics of the CFMT scores.

#### Experience questionnaire

Participants completed a 2-part questionnaire about their experience with Caucasians and Asians. The questionnaire was based on the individuating experience and social contact questionnaires from Walker and Hewstone (2006). First, the individuating experience scale measured how often participants engaged in activities with Asian/Caucasian individuals (5 items for each race). Second, the social contact scale quantified the interactions with Asian/Caucasian people (5 items for each race).

While both the social contact scale and the individuating experience scale have been used separately in several studies (Bukach et al., 2012; Zhao et al., 2014), here we found that the ratings were positively correlated in our sample (for experience with Asian people: *r*_51_ = 0.55, *p* < 0.001; for experience with Caucasian people: *r*_51_ = 0.46, *p* < 0.001). The participants generally had more social contact and individuating experience with Caucasian than Asian people (*t*_52_ = 6.98, *p* < 0.001, *d* = 0.96; *t*_52_ = 5.81, *p* < 0.001, *d* = 0.80; respectively). Following Walker and Hewstone (2006), we averaged the experience scores between the two scales for the analysis. Table 1 shows the descriptive statistics of the experience scores.

## Results

**Fig. 1:**
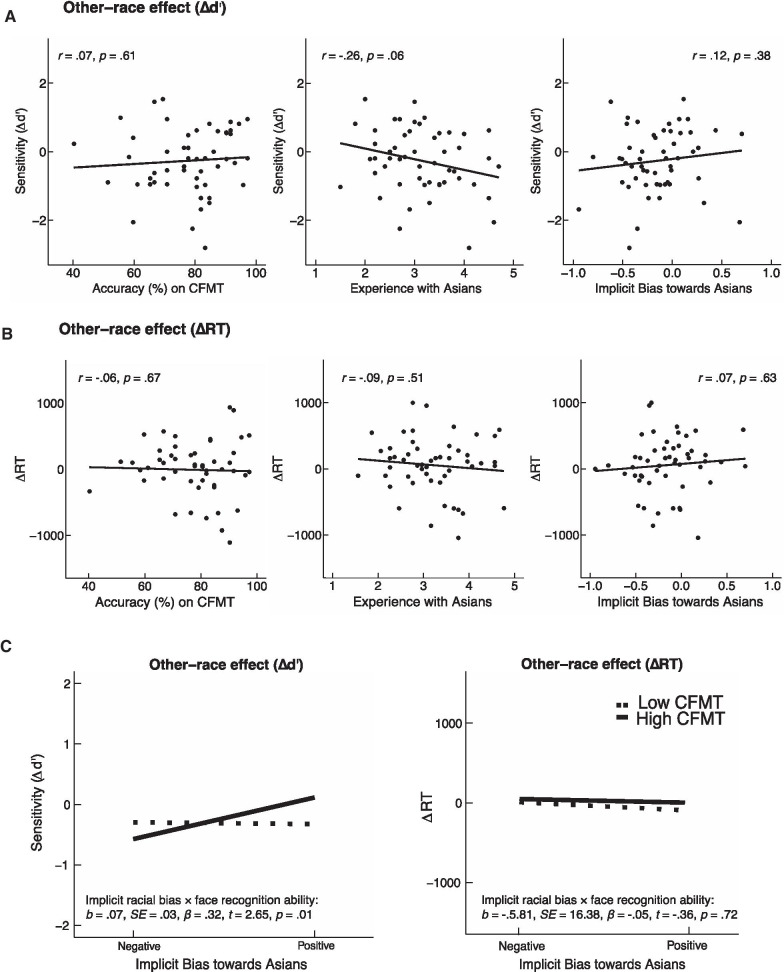
The other-race effect in the memory task. **A**, **B** Scatterplots (with best-fitting regression lines) showing the relationship between the ORE with face recognition ability, experience, and implicit bias, in ∆d’ and ∆RT. **C** Mean other-race effect (∆d’ and ∆RT) as a function of 1 SD above and below the means of the measures of implicit racial bias (IAT) and face recognition ability (CFMT). The ORE in ∆d’ was predicted by experience, implicit racial bias, and the interaction between implicit racial bias and face recognition ability. For ∆RT, neither the regression model, nor any of the predictors, were significant

**Fig. 2:**
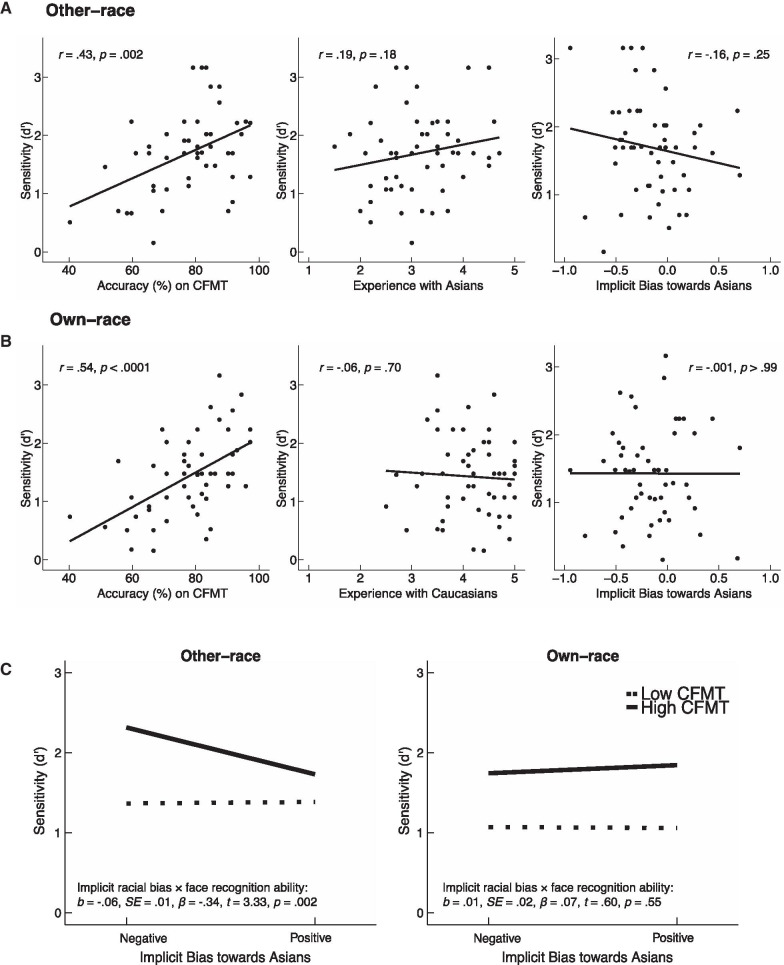
Memory performance for other- and own-race faces. **A**, **B** Scatterplots (with best-fitting regression lines) showing the relationship between sensitivity (d’) in the memory task with face recognition ability, experience, and implicit racial bias for other-race faces (**A**) and for own-race faces (**B**). **C** Mean sensitivity (d’) as a function of 1 SD above and below the means of the measures of implicit racial bias (IAT) and face recognition ability (CFMT). For other-race faces, performance (d’) was predicted by all three factors (experience, implicit racial bias, and face recognition ability), and the interaction between implicit racial bias and face recognition ability. For own-race faces, d’ was only predicted by face recognition ability

**Fig. 3:**
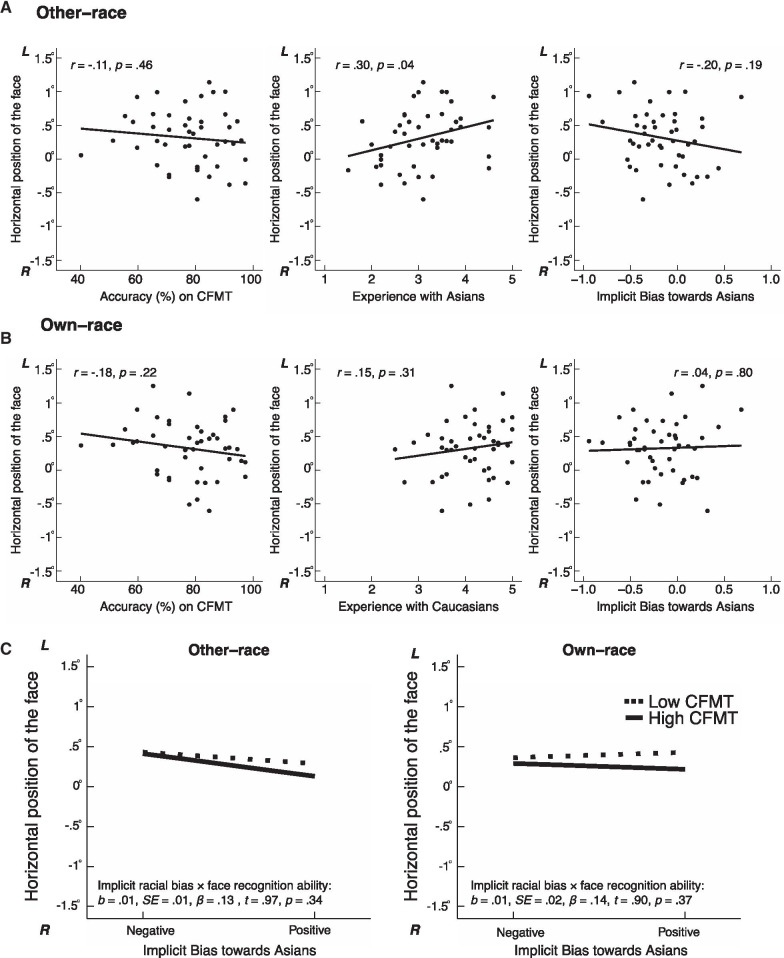
Horizontal position of the first fixation. **A**, **B** Scatterplots (with best-fitting regression lines) showing the relationship between horizontal position (in degrees of visual angle) of the first fixation with face recognition ability, experience, and implicit racial bias for other-race faces (**A**) and for own-race faces (**B**). **C** Mean horizontal positions of the first fixation for other- and own-race faces as a function of 1 SD above and below the means of the measures of implicit racial bias (IAT) and face recognition ability (CFMT). 0°: the center of the face. L: towards the left side of the face; R: towards the right side of the face. For other-race faces, the horizontal position of the first fixation was positively predicted by experience with the other race, and negatively predicted by implicit racial bias. For own-race faces, the regression model was not significant

**Fig. 4:**
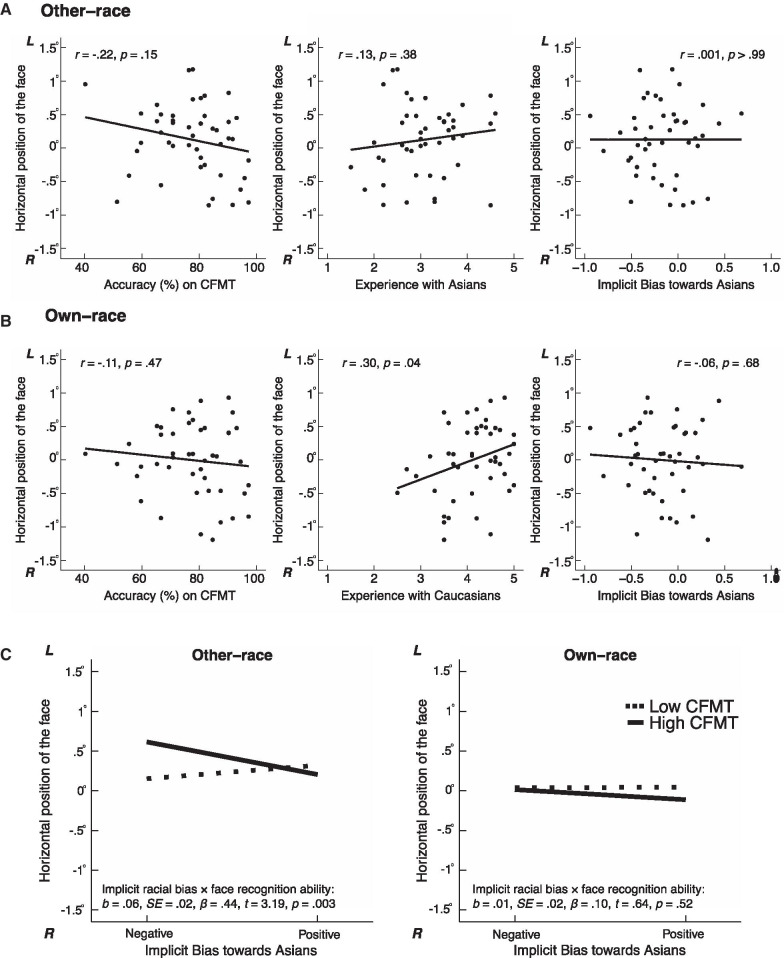
Horizontal position of the second fixation. **A**, **B** Scatterplots (with best-fitting regression lines) showing the relationship between horizontal position (in degrees of visual angle) of the second fixation with face recognition ability, experience, and implicit racial bias for other-race faces (**A**) and for own-race faces (**B**). **C** Mean horizontal positions of the second fixation for other- and own-race faces as a function of 1 SD above and below the means of the measures of implicit racial bias (IAT) and face recognition ability (CFMT). 0°: the center of the face. L: towards the left side of the face; R: towards the right side of the face. For other-race faces, the horizontal position of the second fixation was predicted by the interaction between implicit racial bias and face recognition ability. For own-race faces, the regression model was not significant

We first report the regression results with the factors implicit racial bias, experience, and face recognition ability on memory performance for the ORE, and for other- and own-race faces. We then focus on the eye movements during the learning session and report the regression results on the positions of two initial fixations made on other- and own-race faces. Because of the complexity of the data, we also included the scatterplots between each of the three factors and each of the measures (Figs. 1, 2, 3, 4).^1^ Data analyses were conducted in R version 3.6.1 (Team R Core, 2016).

## Memory performance

### Regression analysis on the other-race effect

For the other-race effects measured in ∆d’ (Caucasian d’ minus Asian d’) and ∆RT (Asian RT minus Caucasian RT), we conducted two separate fixed-effects multilevel regression models, with a random intercept with the three main factors implicit racial bias, experience with the other race, face recognition ability, and the interaction between implicit racial bias and face recognition ability.^2^ All predictors were mean-centered. The scatterplots of ∆d’ and ∆RT with each of the three main factors are illustrated in Fig. 1A, B to show the zero-order correlations. The regression results are shown in Fig. 1C.

The regression model in ∆d’ was significant, *F*_4,48_ = 3.77, *p* = 0.01, *adjusted R*^*2*^ = 0.18. As expected, experience with the other race negatively predicted the ORE, *b* = -0.43, SE = 0.17, *β* = − 0.36*, t* = − 2.63, *p* = 0.01, revealing a reduced ORE with increased experience. Face recognition ability did not predict the ORE, *b* = 0.003, SE = 0.01, *β* = 0.04, *t* = 0.35, *p* = 0.73, but implicit racial bias positively predicted the ORE, *b* = 0.82, SE = 0.39, *β* = 0.29, *t* = 2.11, *p* = 0.04. Importantly, the interaction between implicit racial bias and face recognition ability positively predicted the ORE, *b* = 0.07, SE = 0.03, *β* = 0.32, *t* = 2.65, *p* = 0.01. Follow-up analyses revealed that observers with low face recognition ability did not appear to be influenced by implicit racial bias, *b* = − 0.08, SE = 0.50, *β* = − 0.03, *t* = − 0.16, *p* = 0.88, but observers with high face recognition ability who had increased negative implicit biases towards people in the other race showed a reduced ORE, *b* = 1.72, SE = 0.54, *β* = 0.61, *t* = 3.21, *p* = 0.002.

The regression model in ∆RT was not significant, *F*_4,48_ = 0.36, *p* = 0.84, *adjusted R*^*2*^ = − 0.05.

### Regression analysis on memory of other- and own-race faces

To further examine whether the effects on the ORE in d’ were driven by performance for other- or own-race faces, we conducted two fixed-effects multilevel regression models, separately on other- and own-race faces, with a random intercept with the three main factors and the interaction between implicit racial bias and face recognition ability.^3^ The scatterplots of d’ and each of the three main factors are illustrated in Fig. 2A, B. The regression results are shown in Fig. 2C.

#### Other-race faces

The regression model was significant, *F*_4,48_ = 9.03, *p* < 0.001, *adjusted R*^2^ = 0.38. Experience with the other race, *b* = 0.31, SE = 0.11, *β* = 0.33, *t* = 2.76, *p* = 0.01, and face recognition ability, *b* = 0.03, SE = 0.01, *β* = 0.45, *t* = 4.14, *p* < 0.001, both positively predicted memory performance for other-race faces. Critically, the main effect of implicit racial bias, *b* = − 0.71, SE = 0.26, *β* = -0.32, *t* = − 2.73, *p* = 0.01, and the interaction between implicit racial bias and face recognition ability *b* = − 0.06, SE = 0.02, *β* = − 0.34, *t* = − 3.33, *p* = 0.002, negatively predicted memory performance for other-race faces. Follow-up analyses revealed that observers with low face recognition ability did not appear to be influenced by implicit racial bias, *b* = 0.04, SE = 0.33, *β* = 0.02, *t* = 0.13, *p* = 0.89, but observers with high face recognition ability who had increased negative implicit biases towards people in the other race showed better memory performance for the other-race faces, *b* = − 1.46, SE = 0.36, *β* = − 0.67, *t* = − 4.09, *p* < 0.001.

#### Own-race faces

The regression model was significant, *F*_4,48_ = 5.10, *p* = 0.002, *adjusted R*^2^ = 0.24. In contrast with the results for other-race faces, only the main effect of face recognition ability was significant, *b* = 0.03, SE = 0.01, *β* = 0.54, *t* = 4.46, *p* < 0.001, with higher CFMT scores positively predicted memory performance for the own-race faces. There were no other significant results (*p*’s > 0.55). Indeed, the best-fitted model for own-race faces only included the single predictor of face recognition ability, *F*_1,51_ = 21.13, *p* < 0.0001, *adjusted R*^2^ = 0.28.

## Eye movements

For the analysis, we first removed the trials with fixations coinciding with the stimulus onset, or extreme outliers (< 60 ms or > 1200 ms fixation duration). Data from 7 participants with excessive numbers of removed trials (> 8%) for those fixations were excluded from the analysis. The final data set consisted of 1242 (out of 1288) of the first fixations and 1254 (out of 1288) of the second fixations made during learning other- and own-race faces, with 3.57% and 2.64% of data exclusion for the first and second fixations, respectively. The analyses were performed on the degrees of visual angle from the center of each face.

### Regression analysis on initial fixation positions for other- and own-race faces during learning

Similar to the analysis on memory performance, we conducted four fixed-effects multilevel regression models, separately on the first or second fixations for either other-race or own-race faces, with a random intercept with the three main factors, and the interaction between implicit racial bias and face recognition ability, to predict the horizontal position of the two initial fixation positions during the learning of either other- and own-race faces.^4^ The scatterplots of the fixation positions and each of the three main factors are illustrated in Fig. 3A, B for the first fixation, and in Fig. 4A, B for the second fixation. The regression results are shown in Fig. 3C for the first fixation and in Fig. 4C for the second fixation.

#### Other-race faces

For the first fixation position during the learning of other-race faces, the regression model was significant, *F*_4,41_ = 3.09, *p* = 0.03, *adjusted R*^2^ = 0.16. The main effect of experience was significant, *b* = − 0.25, SE = 0.09, *β* = − 0.44, *t* = − 2.96, *p* = 0.01*,* where participants with more experience with the other-race showing first fixations towards the left side of the other-race faces. The significant main effect of implicit racial bias, *b* = 0.53, SE = 0.20, *β* = 0.41, *t* = 2.65, *p* = 0.01, revealed that participants with a negative bias towards Asians made their first fixations further towards the left side of the faces. There was neither a significant main effect of face recognition ability, nor a significant interaction between implicit racial bias and face recognition ability (*p*’s > 0.33).

For the second fixation position, the regression model was also significant**,** *F*_4,41_ = 3.44, *p* = 0.02, *adjusted R*^2^ = 0.18. A significant interaction between implicit racial bias and face recognition ability was observed, *b* = 0.06, SE = 0.02, *β* = 0.44, *t* = 3.19, *p* = 0.003. Similar to the memory performance for other-race faces, implicit racial bias appeared to only affect participants with high recognition ability, *b* = 1.003, SE = 0.37, *β* = 0.62, *t* = 2.70, *p* = 0.01, but not those with low recognition ability, *b* = -0.40, SE = 0.29, *β* = − 0.25, *t* = − 1.37, *p* = 0.18. Specifically, those with high face recognition ability who had negative implicit biases towards the other race appeared to place their second fixations towards the left-side of the faces, potentially contributing to their better recognition performance for the other-race faces. No other effect of predictor or interaction showed significant results (*p*’s > 0.11).

#### Own-race faces

For either the first or second fixation position during the learning of own-race faces, the regression models were not significant (first fixation: *F*_4,41_ = 0.78, *p* = 0.55, *adjusted R*^2^ = − 0.02; second fixation: *F*_4,41_ = 1.27, *p* = 0.30, *adjusted R*^2^ = 0.02; respectively).

## Discussion

The main goal of the study was to investigate the role of implicit racial bias, in addition to the influences of experience and face recognition ability, on the ORE and specifically on recognition of other-race faces. We tested a group of Caucasian (White) participants who lived in a highly multicultural city and had frequent social interactions with members from various racial groups in the community. Consistent with previous findings (e.g., Hancock & Rhodes, 2008; Walker & Hewstone, 2006; Zhao et al., 2014), the present study revealed a significant effect of experience on the individual differences in the ORE. Specifically, we found that an increase in experience with Asians predicted a reduced ORE, with an improvement in memory performance for other-race faces. Additionally, we showed that an increase in experience with Asians also predicted a stronger left-side bias for Asian faces, as indicated by the horizontal position of the first fixation.

More importantly, although there was an overall negative bias towards Asian people across our Caucasian participants, there was a wide distribution of implicit biases towards Asians ranging from positive to negative biases among the participants. This variability allowed for the study of individual differences of the effect of implicit racial bias on the processing of other-race faces. It is important to note that the effect of implicit racial bias on recognition of other-race faces was independent of that of experience. Although the measures of implicit racial bias and experience were correlated in this study, we found no multicollinearity issue among the factors in the regression analyses. Therefore, these factors independently contributed to the ORE, and more specifically to the memory performance and eye movements for other-race faces. We found that the effect of implicit racial bias was largely dependent on face recognition ability: Caucasian participants with high face recognition ability with a negative implicit bias towards Asians showed the highest recognition performance for Asian faces. The high recognition performance was also accompanied by a strong left-side bias for the second fixation.

The interaction between implicit racial bias and face recognition ability suggests that this socio-cognitive factor does not automatically influence recognition performance of other-race faces. The lack of influence of implicit racial bias for participants with low face recognition ability suggests different levels of recognition processes. With low face recognition ability, participants might only be able to process the available perceptual features at their best capacities, but not to utilize the social features of the faces, such as the information of race. For these participants, the initial eye movements of participants when processing the other-race faces also did not appear to be influenced by implicit racial biases, presumably due to perceptual limitations in the encoding of diagnostic features for recognition. In contrast, the effect of implicit racial bias was observed only in participants with high face recognition ability, suggesting that social features of the faces were also incorporated in the recognition processes. It is possible that participants with high face recognition ability had larger perceptual or cognitive capacities to process additional information beyond perceptual features during recognition of other-race faces. Although the memory task required individuation, these participants showed evidence that the other-race faces were also categorized by their race (Levin, 1996, 2000). Importantly, it appears that the implicit biases towards individuals of that race, affected how the other-race faces are encoded and remembered in participants with high face recognition ability.

Our finding showed that a negative, but not a positive, implicit bias towards another race led to superior memory performance for the other-race faces in the participants with high face recognition ability. This result was unexpected, as positive implicit biases towards the other-race, along with increased individuating experience with the other race, have shown to be related to improved perceptual discrimination of other-race faces (Walker & Hewstone, 2008), and enhanced recognition performance for other-race faces (Lebrecht et al., 2009). There are several possible explanations for these differences. It is possible that implicit racial biases might have different influences on perception and memory of other-race faces. Moreover, the relationship between individuating experience and implicit racial biases was not distinguished in these previous studies, and thus it is difficult to identify whether the enhanced perceptual or memory performance was due to either increased individuating experience or positive implicit biases with the other race. On the one hand, our results showed that an increase in experience with the other race reduced the magnitude of the ORE and improved memory performance of faces from that race. On the other hand, we found that independent of the effect of experience, an increase in negative implicit racial biases led to superior memory performance of other-race faces in participants with high face recognition ability. The improved memory performance of other-race faces in the participants with high face recognition ability and negative implicit racial bias was accompanied by the left-side bias, a hallmark of successful face recognition revealed by eye movements, with the second fixations positioned towards the left side of the faces.

One possibility for this effect of implicit racial bias may involve enhanced attention to the individual faces of the other race. It has been shown that implicit negative bias prompts observers to more readily perceive other-race faces with ambiguous expressions to be angry (Hugenberg & Bodenhausen, 2003), and angry expressions could enhance recognition performance of other-race faces (Ackerman et al., 2006; Young et al., 2012). Although only faces with neutral expressions were used in this study, participants with high recognition ability and negative implicit bias might still allocate additional attention to the individual other-race faces for successful recognition. Future research should further investigate how the encoding of race information, and the perception of anger or threat arises from negative implicit racial biases, may lead to improved recognition of individual faces from other races.

We note that with regard to the effect of experience on other-race face recognition, previous studies had emphasized the potential differences between individuating experience and social contact experience in face recognition: individuation experience appears critical to improve perceptual discrimination or enhance holistic processing of other races (e.g., Bukach et al., 2012; Walker & Hewstone, 2008), whereas social contact experience has been found to be positively related to memory performance of other-race faces (Zhao et al., 2014). Nonetheless, in our Caucasian participants who had relatively extensive experience with Asians, the scores obtained from the two experience scales were positively correlated (see also Walker & Hewstone, 2006). Since both scales produced highly similar results on memory performance and eye movements for both other- and own-race faces, we combined the two scales as one measure of experience. It is possible that while these two types of experience are potentially related to different aspects of face processing, the differences between these two types of interactions might have been minimized when participants had extensive experience with the other race.

While the influences of implicit racial biases and individuating/social contact experience on the recognition of other-race faces were observed, these factors did not appear to affect the recognition of own-race faces. Instead, the performance of own-race faces was only predicted by the participants’ face recognition ability. It is possible that the extensive experience participants had with members of their own race led to a limited range of experience scores for predicting recognition performance or initial eye movements for own-race faces. Moreover, implicit biases towards another race also did not appear to influence recognition or eye movements of own-race faces, suggesting that the socio-cognitive evaluation of implicit racial biases did not automatically apply to any faces, but it might only be specifically activated for other-race faces.

In terms of potential limitations, we acknowledge that our Caucasian participants were from various countries. Because there are slight variations in the appearance of Caucasians from different regions (e.g., Northern Europeans/Australians/Germans or Southern Europeans/Americans, Bowles et al., 2009; Chiroro et al., 2008; McKone et al., 2011, 2012), the face stimuli used in the CFMT or memory task in this study might not best match the ‘own-race’ group of all of our Caucasian participants. Nonetheless, it is likely that Caucasian participants identified those faces as from their own group, as the ‘other-ethnicity’ effect is not always consistently observed (McKone et al., 2012; Sporer, 2001; Sporer et al., 2007), and a reliable ORE could be found in Caucasian participants who had relatively little experience with Asians, with the same version of the CFMT that might not most optimally matched their own ethnicity (e.g., Germans, Zhao et al., 2014). Therefore, while some additional variabilities might have been introduced in the data, the version of the CFMT used in this study was still likely valid to measure face recognition ability in our Caucasian participants, and to predict their recognition performance of other- and own-race faces.

Moreover, although this study only included Caucasian participants, the main goal here was not necessarily to demonstrate a general effect of implicit racial bias on recognition of other-race faces. There are challenges to conduct this type of research using participants across different races, as the effect of individual differences requires a range of variability in the data. We were able to obtain a range of positive and negative implicit biases towards Asians in our Caucasian participants in this study. However, because of the generally favorable implicit biases towards Caucasians, and generally negative implicit biases towards Black/Africans, it is unfortunately very challenging to obtain a good range of data for implicit racial biases for any particular races. We hope that the increase in multicultural exchanges would lead to understanding and appreciation of the qualities about different races, which may also benefit the research on implicit racial biases on face recognition.

## Conclusions

The present study shows that in a group of Caucasian participants in a multicultural city who had relatively extensive experience with Asians, recognition of Asian faces was independently predicted by experience and implicit racial bias. While recognition was improved with increased experience with the other race, it was also improved in participants with high face recognition ability and negative implicit biases towards Asians. Because the effects of experience and implicit racial bias did not affect recognition performance of Caucasian faces, these results suggest that the processing of social features of a face, such as race, is not automatic but it is incorporated by participants with high face recognition ability during the processing of other-race faces.

### Significance statement

In the age of globalization, there are increased interactions among social and racial groups. The multicultural and multiracial interactions encourage understanding among groups, but there are also potential new conflicts. For everyday interactions, faces convey much useful information for identification and social evaluation. The current investigation of individual differences revealed that better recognition performance for the other-race faces was positively related to an increase in experience with the other-race and with negative implicit racial biases in participants with high face recognition ability. These effects of experience, implicit racial bias, and face recognition ability were also supported by the initial eye movements when learning the other-race faces. We suggest that the information of race is evaluated and incorporated involuntarily in the recognition processes by participants with high face recognition ability, but it may not be utilized by participants with low face recognition ability. These findings suggest the complexity in understanding the perceptual and socio-cognitive influences on recognizing faces of other races.

## Data Availability

The dataset and analysis code of the study are available from the OSF website: https://osf.io/etjcs/?view_only=470f9592fbd440d3aacadbad16ffbc69. The files include the regression analyses reported in the main text, and additional group-level results for the memory task and for the eye movement data. The dataset and analysis code of an additional online study are also available on the same website, with the group-level comparisons of the memory performance between self-reported monocultural Asian and Caucasian participants, and between the Caucasian participants tested online and in the lab.

## Abbreviations

ORE: Other-race effect
CFMT: Cambridge face memory test
IAT: Implicit association test

## Footnotes

1 As online supplemental materials, we also included additional group-level analyses on the memory and eye movement data of this study, and group-level comparisons of the memory performance between additional groups of self-reported monocultural Asian and Caucasian participants, and between Caucasian participants in this study and the self-reported monocultural Caucasian participants.

2 The model with three main factors and the interaction between IAT and CFMT was the best model as indicated by stepwise regression analysis. Specifically, including additional interaction terms did not significantly improve the model fit for the ORE in the memory task in ∆d’ (*p* = .643) or ∆RT (*p* = .335). Among the three main factors, there was a positive correlation between the implicit racial bias scores and the experience scores towards Asians (*r* = .386*, p* = .004). Nonetheless, the Variance Inflation Factor (VIF) estimates suggested that there were no multicollinearity issues among any of the factors and their interactions (VIF_IAT_ = 1.28; VIF_CFMT_ = 1.21; VIF_Experience: Asians_ = 1.30; VIF_IAT_:VIF_CFMT_ = 1.19).

3 The stepwise regression analysis for either other- or own-race faces revealed no significant improvement in the model fit when additional interaction terms were included in the model (*p*’s > .201).

4 The stepwise regression analysis revealed no significant improvement in the model fit when additional interaction terms were included in the model for either the first or second fixations on either other- or own-race faces (*p*’s > .212).
